# A Cooperative Merging Method for Mixed Traffic Based on Enhanced Graph Reinforcement Learning with Vehicle Collaboration Graphs

**DOI:** 10.3390/s26041225

**Published:** 2026-02-13

**Authors:** Haifeng Guo, Hongda Fu, Dongwei Xu, Tongcheng Gu, Enwen Qiao, Baiyang Ji

**Affiliations:** 1 Institute of Cyberspace Security, Zhejiang University of Technology, Hangzhou 310023, China; guohf@zjut.edu.cn (H.G.); 211123030073@zjut.edu.cn (H.F.); 121123030036@zjut.edu.cn (T.G.); 211123030143@zjut.edu.cn (E.Q.); 2 College of Computer Science and Technology, Zhejiang University of Technology, Hangzhou 310023, China; jby@zjut.edu.cn

**Keywords:** connected and autonomous vehicles, cooperative decision-making, ramp merging, Graph Reinforcement Learning, mixed-autonomy traffic

## Abstract

Achieving cooperative perception and decision-making among connected and autonomous vehicles (CAVs) in mixed-traffic ramp merge scenarios is crucial for building a swarm intelligence-based traffic control system. However, existing cooperative decision-making methods struggle to adequately model and represent the dynamic collaborative interactions among heterogeneous agents in mixed-traffic environments, which can lead to traffic congestion or even severe accidents in ramp merging areas. Therefore, this paper proposes an Enhanced Graph Reinforcement Learning algorithm based on a Vehicle Collaboration Graph (VCG-EGRL) to enable cooperative merging decisions for CAVs in mixed-traffic ramp merging scenarios. First, a vehicle collaboration intensity (VCI) model is designed to effectively model the intensity of collaborative interactions among vehicles. Then, based on the VCI model, the perception–communication relationships between vehicles and the vehicle-to-infrastructure (V2I) communication relationships are jointly constructed to form a local–global cooperative graph, which represents the dynamic collaborative relationships of the vehicle network from macro and micro perspectives and deeply explores the driving behavior of vehicles. Subsequently, a Graph Convolutional Network enhanced with Kolmogorov–Arnold Networks (KANs), referred to as GKAN, is employed to extract and aggregate the driving features of vehicles from the local–global graph. Finally, a graph mutual information maximization method is used to optimize the iterative process of the Graph Reinforcement Learning strategy, ensuring the generation of accurate lane-changing decisions for CAVs. Experimental results in ramp merging scenarios under varying traffic conditions demonstrate that the proposed method outperforms baseline models in terms of merging success rate, efficiency, and robustness.

## 1. Introduction

Ramp Merging Zones are critical and interaction-intensive traffic nodes in road networks. Since vehicles must coordinate speed, lane selection, and space allocation during merging, these areas are prone to traffic congestion and even gridlock [1]. Meanwhile, with the advancement of autonomous driving technology, road systems will operate in a mixed traffic environment where human-driven vehicles (HDVs) and CAVs coexist. Therefore, a key challenge for modern intelligent transportation systems is to effectively control CAVs in mixed-traffic Merging Zones, enabling cooperative perception and driving while completing relevant driving tasks and improving the overall operational efficiency and safety of the traffic system [2].

Many methods have been proposed by researchers to address the ramp merging problem. Rule-based approaches can effectively improve traffic efficiency in simple scenarios, but their applicability and effectiveness significantly decrease in more complex ramp merging situations under mixed-traffic conditions [3]. Although optimization-based methods can ensure the safety of the decision-making process, their high computational demands often hinder real-time applicability in dynamic traffic scenarios [4].

In contrast, data-driven methods such as deep reinforcement learning (DRL) have attracted growing attention for their adaptive learning without explicit modeling and have been applied to CAV control in merging scenarios [5]. DRL-based models leverage the powerful function approximation capability of deep neural networks to efficiently capture and represent state information from the environment, and rapidly establish mappings from states to actions, thereby enabling the formulation of optimal decision-making strategies [6].

Currently, DRL approaches for enabling multiple CAVs to cooperatively complete merging tasks safely and efficiently can be categorized into two main control strategies: centralized control and distributed control [7]. Centralized control strategies utilize Vehicle-to-Vehicle (V2V) and V2I communication technologies to establish interaction and cooperation mechanisms between vehicles and road infrastructure, thereby forming an integrated vehicle–road cooperative control system [8]. Centralized control typically assumes that all CAVs within the cooperative system are communication-enabled and rely on infrastructure such as Roadside Units (RSUs) for centralized decision-making and computation, which imposes high demands on the system’s real-time communication capabilities. Roadside traffic control and decision-making based on V2I communication have been regarded as an effective and practical solution for cooperative driving [9]. RSUs make decisions from a global perspective, enabling more comprehensive perception of the traffic environment and more effective optimization of overall system performance compared to the local perspective of autonomous vehicles. In contrast, distributed control methods are primarily implemented through multi-agent reinforcement learning algorithms, where each vehicle in the merging area is treated as an intelligent agent capable of perceiving its environment and making decisions independently. Cooperation among multiple vehicles is achieved through specifically designed coordination policies. However, in distributed control methods, the limited perception range of individual vehicles may result in insufficient information to form a global perspective, which may lead to locally optimal rather than globally optimal decision-making [10]. Therefore, centralized control is considered more suitable for cooperative decision-making among multiple CAVs in complex mixed-traffic merging scenarios.

In mixed-traffic environments, the interactions between CAVs, as well as between CAVs and HDVs, are becoming increasingly complex. Existing centralized reinforcement learning methods typically incorporate only the states of the ego vehicle and nearby vehicles as multidimensional inputs, without sufficiently modeling the intricate interaction relationships among vehicles. In addition, these methods fail to extract environmental information effectively. Such limitations may lead to degraded performance of cooperative driving strategies for CAVs under mixed-traffic conditions. Graph representation learning can explicitly characterize objects and their interactions, offering strong expressive power, and has been applied in areas such as game analytics and prediction, social network analysis, and recommender systems. For example, Melo et al. [11] proposed and validated a graph representation learning approach based on game provenance causal graphs for game event/outcome prediction, which demonstrates advantages over methods relying on handcrafted game metrics in certain tasks. Therefore, recent studies have proposed a centralized control framework based on Graph Reinforcement Learning (GRL) [12] for designing cooperative decision-making systems for CAVs. Compared to conventional DRL-based centralized approaches, this method integrates graph representation learning and Graph Neural Networks (GNNs) into the DRL framework, enabling the modeling of interactions among vehicles and thereby achieving superior performance in cooperative decision-making tasks. Despite these advantages, most existing GRL-based centralized control approaches still face several limitations: (1) The modeling of dynamic interactions between vehicles is often overly simplistic or limited, typically focusing only on communication-based relationships. (2) The current use of GNNs for feature extraction in GRL remains insufficient. Accurately understanding and effectively learning environmental states is a key component of such reinforcement learning methods and directly affects overall model performance. (3) In recent years, research in robotics has increasingly focused on improving the stability and efficiency of reinforcement learning training. For example, Nguyen et al. [13] proposed a policy-iteration-integrated online and offline model-free Q-learning control approach, which enhances learning efficiency and control performance in a two-wheeled inverted pendulum task with uncertain dynamics by decoupling the system into subsystems and analyzing the effect of probing noise. However, most existing GRL algorithms are trained solely using reinforcement learning loss functions. However, when dealing with complex tasks such as ramp merging in mixed traffic, it is necessary to learn behavior policies that are closely aligned with environmental feedback (i.e., rewards). This single-objective training mechanism may fail to fully capture the underlying relationship between input graph embeddings and output Q-values, potentially overlooking critical details relevant to the target behavior, thereby limiting the overall effectiveness of vehicle control policy optimization.

In addition, current research primarily emphasizes local control of vehicles within the Merging Zone or remains limited to single-lane scenarios. Due to insufficient control over vehicles in the ramp preparation zone, road resources are not fully utilized, hindering the development of more systematic and cooperative vehicle control strategies.

Therefore, this paper proposes a novel framework named VCG-EGRL to effectively address the cooperative decision-making problem of vehicles in ramp Merging Zones under mixed-traffic conditions. The proposed method enables the control of CAVs to successfully complete merging tasks while ensuring the overall smoothness of traffic flow. The main contributions of this paper are summarized as follows:

(1) An Enhanced Graph Reinforcement Learning network architecture is proposed for multi-agent cooperative ramp merging and lane-changing decision-making in mixed-traffic environments. The architecture builds a local–global collaboration graph based on a vehicle collaboration intensity model to deeply capture vehicles’ spatial dynamic interactions, and enables optimal merging decisions through Graph Reinforcement Learning.
(2) A training framework for GRL is proposed based on graph mutual information maximization, which integrates a designed graph mutual information loss with the Deep Q-network (DQN) loss to jointly train the entire algorithm, improving Q-value prediction accuracy and enhancing the performance of the Graph Reinforcement Learning algorithm, thereby generating accurate cooperative control strategies for vehicles.
(3) An assisted merging lane-changing strategy is proposed for vehicles in the ramp Pre-Merging Zone, which actively guides CAVs in real time to perform proactive lane changes to fully utilize lane capacity, increase the number of available merging gaps, reduce traffic conflicts between ramp and mainline vehicles, and improve the overall traffic efficiency and safety of the merging area.
(4) Based on the above three components, we propose a cooperative decision-making algorithm for ramp merging in mixed traffic, named VCG-EGRL. Experiments conducted under varying CAV penetration rates and mainline traffic densities show that VCG-EGRL outperforms state-of-the-art baselines across multiple metrics.

## 2. Related Work

This section reviews two main categories of ramp merging strategies under mixed traffic. The first focuses on optimization-based approaches, and the second on DRL-based approaches.

### 2.1. Optimization-Based Ramp Merging Strategies

To plan the trajectories or merging sequences of CAVs in mixed-traffic environments, Zhao et al. [14] propose a macro–micro coordinated ramp merging control framework: at the macroscopic level, MPC is used to optimize variable speed limits and ramp metering; at the microscopic level, virtual platooning coordinates AV trajectories to enable signal-free cooperative merging, thereby alleviating congestion and reducing travel time. Zhang et al. [15] propose a merging method based on platoon coordination and downstream gap redistribution, where mainline CAV speed guidance reserves merging space for on-ramp platoons, improving merging efficiency while reducing energy consumption. Jiang et al. [16] propose a two-layer ramp merging control framework based on a Stackelberg game, integrating real-time driving behavior estimation with trajectory optimization to enable more efficient and energy-saving cooperative merging of CAVs in mixed traffic. Yang et al. [17] proposed a safety-oriented merging method based on a bi-level gap selection function, which reduces collision risk for CAVs and determines the safest merging sequence. Hou et al. [18] proposed a hierarchical distributed merging control model (CORMC) for multi-lane mixed traffic, which integrates high-level policy planning with low-level merging execution to improve merging efficiency. Liu et al. [19] proposed a safe and flexible hierarchical cooperative ramp merging control strategy, in which the upper layer determines the target merging position, while the lower layer performs trajectory optimization to generate time-efficient and energy-efficient trajectories for CAVs. Li et al. [20] proposed DCoMA, a dynamic cooperative ramp merging strategy that provides macroscopic platoon guidance and microscopic ramp-vehicle control to improve merging efficiency and safety.

However, the above ramp merging strategies often rely on precise mathematical modeling of vehicle dynamics and the environment, leading to high computational costs and potential real-time delays in practical applications. Moreover, such methods are prone to local optima in long-horizon trajectory planning.

### 2.2. DRL-Based Ramp Merging Strategies

In contrast, DRL methods have shown great potential in addressing complex decision-making problems in high-dimensional environments [21]. With well-designed state and action spaces and a properly defined reward function, DRL-based CAV driving algorithms can outperform human drivers. As a result, recent studies have explored applying DRL to CAV decision-making in ramp merging scenarios under mixed-traffic conditions. For example, Cheng et al. [22] proposed a learning-based vehicle sequencing framework that combines Transformer-based spatiotemporal encoding with PPO reinforcement learning, and improves traffic efficiency in simulation. Kherroubi et al. [23] proposed a DRL-based control method integrated with intention recognition, which uses neural networks to predict HDV intentions and enables safe and cooperative merging decisions. Chen et al. [24] proposed a multi-agent reinforcement learning (MARL) control framework with a priority-based safety supervisor, which significantly reduces collision rates through safety supervision. Li et al. [25] proposed an interactive merging strategy based on MARL, which models the competition between mainline and ramp vehicles as a game and guides CAVs to make optimal responses by solving the Nash equilibrium. Liu et al. [26] proposed a ramp merging method that integrates DRL with a motion-prediction-based safety controller, where a trajectory prediction and action replacement module is used to reduce collision risk during merging. Zhang et al. [27] proposed an Independent Proximal Policy Optimization (IPPO) algorithm to improve multi-agent decision success rates in highway ramp merging for autonomous vehicles. However, these methods mainly focus on controlling single or a small number of agents, with limited observation and action spaces. Moreover, most of them fail to fully model the complex interactions among vehicles, which may lead to poor coordination and unstable performance in more complex traffic scenarios, hindering optimal decision-making. Lin et al. [28] proposed KL-MA2C, which integrates a KAN–LSTM module into multi-agent A2C and incorporates TTC constraints and MPC-based smoothing control to improve the safety and efficiency of ramp merging.

To overcome the limitations of the above methods, recent studies have explored GRL-based approaches for multi-CAV cooperative decision-making, demonstrating superior performance over traditional DRL methods. For example, Chen et al. [29] proposed an algorithm combining Graph Convolutional Networks (GCNs) with DQN, where the traffic network is modeled as an undirected graph, GCN is used for feature extraction, and DQN generates lane-changing decisions for CAVs in ramp scenarios. Wang et al. [30] proposed a Spatiotemporal Deep Q-Network (STDQN) framework that integrates a Double DQN with a spatiotemporal feature extraction module to effectively capture the spatiotemporal information of CAVs. Liu et al. [2] proposed a Graph Convolutional Proximal Policy Optimization (GCAV-CPO) framework that integrates vehicle graph structures with MARL to enable safe and eco-friendly ramp merging for CAVs in multi-lane environments. Hu et al. [31] proposed a GRL method combining multi-head attention and spatial graph convolution to enable cooperative decision-making among multiple CAVs.

The above GRL methods, represented by GCQ, can address the Dynamic Number of Agents Problem (DNAP), where the number of observed and controlled vehicles varies in the cooperative decision-making process [29]. However, these methods rely solely on perception and communication to establish information flow between CAV nodes, resulting in overly simplistic interaction modeling. Moreover, they fail to effectively utilize information about distant HDVs beyond a single CAV’s perception range, limiting decision-making performance. In addition, these GRL approaches are typically trained using only RL-based loss functions, neglecting potential relationships between graph embeddings and Q-values, which undermines the GNN’s ability to extract critical driving features and weakens overall decision quality. To address these limitations, the proposed VCG-EGRL framework explores vehicle interaction topology from both micro and macro perspectives, fully leveraging the feature extraction capabilities of graph networks to generate more forward-looking and effective cooperative merging decisions. This facilitates efficient multi-vehicle coordination and improves overall traffic efficiency in ramp Merging Zones.

## 3. Problem Settings

### 3.1. Scenario Settings

**Figure 1:**
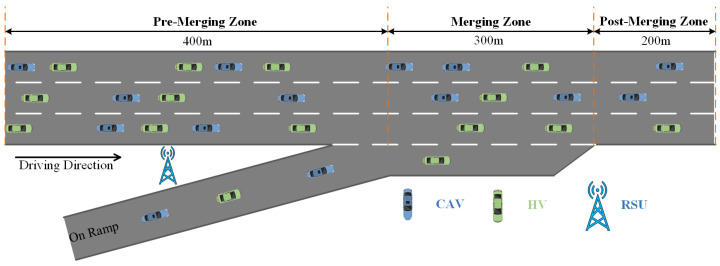
Traffic scenario adopted in this study.

In this study, we use Flow [32], a DRL framework integrated with the urban traffic simulator SUMO [33], to construct a mixed-traffic simulation environment and generate traffic flow. As shown in Figure 1, the scenario consists of a three-lane main road and a single-lane on-ramp. To clearly describe the merging process, the scenario is divided into four zones: the Pre-Merging Zone (upstream segment of the merging area), the on-ramp, the Merging Zone, and the Post-Merging Zone (downstream segment). Blue vehicles represent CAVs, and green vehicles represent HDVs. The initial states of both CAVs and HDVs, such as speed and position, are randomly assigned by SUMO. HDVs are controlled using SUMO’s built-in Intelligent Driver Model (IDM) [34] for longitudinal behavior and the LC2013 model [35] for lane changing. CAVs adopt the IDM model for longitudinal control and use DRL models such as VCG-EGRL, GCAV-CPO, and GCQ for lateral decision-making. An RSU serves as the centralized control entity in the simulation, acting as the DRL agent to coordinate CAVs within the ramp merging area from a global perspective. The DRL controller provides lane-changing decisions for CAVs, allowing mainline CAVs to proactively assist ramp vehicles in merging safely and efficiently. At the same time, it aims to maximize the overall traffic flow efficiency within the Merging Zone.

### 3.2. Problem Definition

The formulations in this section define the MDP and reward structure that provide the learning objective and evaluation basis for the proposed VCG-EGRL framework.

The multi-vehicle cooperative decision-making problem in the mixed-traffic environment is formulated as a Markov Decision Process (MDP) [36], defined by a five-element tuple 〈S,A,R,P,γ〉, where *S* denotes the state space, which is used to describe the current environmental state information; *A* represents the action space; *R* is the immediate reward; *P* represents the state transition probability; and γ∈(0,1] is the discount factor for future rewards. On this basis, this paper further elaborates the specific modeling and design of the state space, action space, and reward function within the mixed-traffic ramp merging scenario.

#### 3.2.1. State Space

At each time step, the vehicle network in the ramp merging scenario is modeled as an undirected graph. Therefore, the state space of the agent at time step *t* is denoted as $S_{t}=\left(X_{t},A_{t}^{\mathrm{local}},A_{t}^{\mathrm{global}},M_{t}\right)$, where $X_{t}$ represents the driving features of vehicle nodes in the graph, $A_{t}^{\mathrm{local}}$ denotes the local adjacency matrix constructed by the cooperation intensity model, vehicle perception, and V2V communication, $A_{t}^{\mathrm{global}}$ represents the global adjacency matrix constructed via V2I communication and the cooperation intensity model, and $M_{t}$ denotes the masking matrix.

(1) Vehicle Driving Feature $X_{t}$: At time step *t*, there are *N* vehicles in the scenario. The driving feature of vehicle *i* includes position, speed, lane, and acceleration, and is represented by a 7-dimensional vector:
  (1) $$x_{t}^{i}=\left(p_{t}^{i},v_{t}^{i},l_{t}^{i},a_{t}^{i}\right)$$
  where $p_{t}^{i}$ is the normalized longitudinal position of vehicle *i* at time step *t*, normalized by the total length of the main road; $v_{t}^{i}$ is the normalized speed of vehicle *i* at time step *t*, normalized by the maximum speed limit; and $l_{t}^{i}$ is the current lane position of vehicle *i* at time step *t*, represented using one-hot encoding. For example, the ramp is encoded as [0,0,0,1], while mainline lanes 0, 1, and 2 are encoded as [0,0,1,0], [0,1,0,0], and [1,0,0,0], respectively; $a_{t}^{i}$ is the normalized acceleration of vehicle *i* at time step *t*, normalized by the difference between its maximum acceleration and maximum deceleration.
(2) Adjacency Matrices $A_{t}^{\mathrm{local}}$ and $A_{t}^{\mathrm{global}}$: The vehicle network in the ramp merging area is modeled as a graph, where the adjacency matrices are used to represent the cooperation and interactions among nodes (i.e., vehicles) in the graph. This method uses a structured form to describe the complex dynamics and interaction relationships among vehicles, thus providing theoretical support for using GNNs to compute inter-vehicle connectivity, information propagation, and motion planning. In Section 4.2.1, the detailed construction methods of $A_{t}^{\mathrm{local}}$ and $A_{t}^{\mathrm{global}}$ based on the VCI model, vehicle perception, and communication will be introduced. To adapt to the dynamic changes in the number of vehicles within the ramp merging area, the dimension of the adjacency matrices is set as N×N, where *N* is the maximum number of vehicles in the merging area. When the number of vehicles in the ramp merging area is less than *N* or when a vehicle exits the Merging Zone, the corresponding elements in the matrix are set to 0. This ensures that the adjacency matrices maintain computational stability while providing good scalability and flexibility.
(3) Masking Matrix $M_{t}$: This study focuses on decision-making control for CAVs within the ramp merging area only. Therefore, to eliminate irrelevant features related to HDVs in the GNN output, a masking matrix is constructed to filter out the embedding features of HDVs. Specifically, based on the ordering of $x_{t}^{i}$ in $X_{t}$, a binary vector of length *N* is generated as the masking matrix $M_{t}$, where entries corresponding to CAV are set to 1, and those corresponding to HDV are set to 0.

#### 3.2.2. Reward Function

The design of the reward function plays a crucial role in training reinforcement learning agents, as its structure directly reflects task objectives and guides agents toward expected behavioral patterns. In the ramp merging scenario, the primary goal of the agent is to control CAVs to safely complete the merging task while improving overall traffic flow efficiency before completing the task. The reward function designed in this study consists of four components: merging reward $R_{m}$, efficiency reward $R_{v}$, collision penalty $P_{c}$, and time-step penalty $P_{s}$. The overall reward function in this study is defined by

(2) $$R=w_{m}R_{m}+w_{v}R_{v}+w_{c}P_{c}+w_{s}P_{s}$$

Among them, $w_{m}$, $w_{v}$, $w_{c}$, $w_{s}$ represent the weight coefficients corresponding to the merging reward, speed reward, collision penalty, and time-step penalty, respectively. The weight coefficients are predefined and fixed, serving to balance the relative importance among task objectives, driving efficiency, and system safety during the learning process. In this study, the merging reward $w_{m}$ is assigned a relatively high weight to ensure that the agent prioritizes learning the key behavioral strategies required for successful merging during training. The specific weight coefficients are finalized through multiple rounds of training and adjustment as $w_{m}=2$, $w_{v}=1$, $w_{c}=0.8$, $w_{s}=0.6$. The specific definitions of $R_{m}$, $R_{v}$, $P_{c}$, and $P_{s}$ are given.

**Figure 2:**
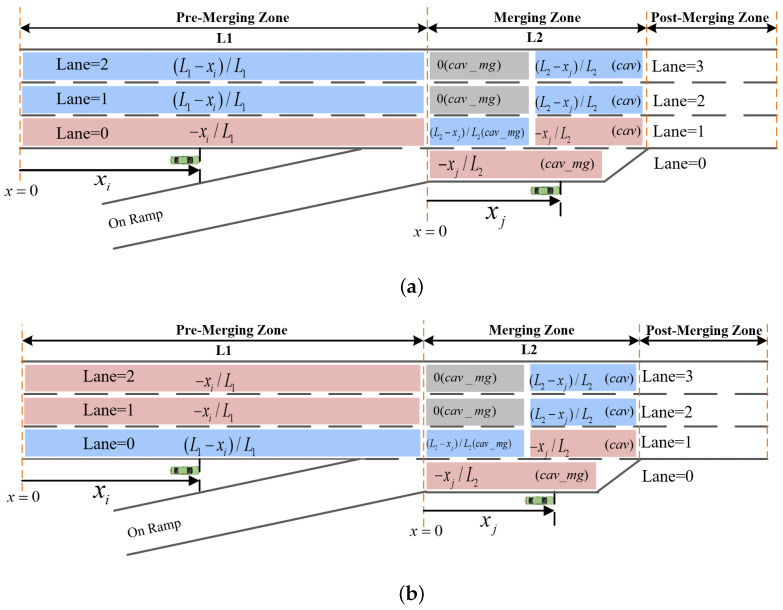
Merging reward diagrams. (**a**) Reward diagram under the condition $D_{rh0\_0}<D_{\mathrm{average}0}$; (**b**) reward diagram under the condition $D_{rh0\_0}>D_{\mathrm{average}0}$.

- $R_{m}$ denotes the merging reward, which is designed to guide CAVs to perform fast and smooth merging maneuvers during the ramp merging process. The corresponding formula is given by
  (3) $$R_{m}=\frac{\sum_{i}^{n}r_{i}+\sum_{j}^{m}r_{j}}{N_{\mathrm{CAV}}}$$
  where $N_{\mathrm{CAV}}$ denotes the number of CAVs in the current scenario, $r_{i}$ denotes the auxiliary merging reward for the *i*-th CAV located in the Pre-Merging Zone. This reward is dynamically adjusted based on the lane the vehicle occupies and the traffic density of that lane. The aim is to encourage vehicles on mainline lane 0 in the Pre-Merging Zone to proactively change lanes based on lane density, thereby creating more merging space for ramp vehicles and reducing congestion risks in the Merging Zone caused by merging conflicts. $r_{j}$ denotes the merging reward for the *j*-th CAV in the Merging Zone. It is designed to encourage ramp vehicles within the Merging Zone to complete merging maneuvers in a fast, safe, and efficient manner. $r_{i}$ and $r_{j}$ are defined as functions of the vehicle’s longitudinal position, so that the reward magnitude for a CAV is determined by its lane-change location. This encourages proactive merging planning and decision-making, thereby improving overall merging efficiency and system robustness.
  $r_{i}$ is calculated as
  (4) $$\begin{array}{cc} \; & D_{rh0\_0}>D_{\mathrm{average}0}:\\ \; & r_{i}=\left\{\begin{array}{cc} -\frac{x_{i}}{L_{1}}, & i\_{\mathrm{laneindex}}_{t}=0\\ \frac{L_{1}-x_{i}}{L_{1}}, & i\_{\mathrm{laneindex}}_{t}=1\;\mathrm{or}\;2 \end{array}\right. \end{array}$$
  (5) $$\begin{array}{cc} \; & D_{rh0\_0}<D_{\mathrm{average}0}:\\ \; & r_{i}=\left\{\begin{array}{cc} -\frac{x_{i}}{L_{1}}, & i\_{\mathrm{laneindex}}_{t}=1\;\mathrm{or}\;2\\ \frac{L_{1}-x_{i}}{L_{1}}, & i\_{\mathrm{laneindex}}_{t}=0 \end{array}\right. \end{array}$$
  (6) $$\begin{array}{cc} \; & D_{rh0\_0}<D_{\mathrm{average}0}:\\ \; & r_{i}=1 \end{array}$$
  $r_{j}$ is calculated as
  (7) $$\begin{array}{c} j\_cavtype=cav\_mg:\\ r_{j}=\left\{\begin{array}{cc} -\frac{x_{j}}{L_{2}}, & j\_{\mathrm{laneindex}}_{t}=0\\ \frac{L_{2}-x_{j}}{L_{2}}, & j\_{\mathrm{laneindex}}_{t}=1 \end{array}\right. \end{array}$$
  (8) $$\begin{array}{c} j\_cavtype=cav:\\ r_{j}=\left\{\begin{array}{cc} & j\_{\mathrm{laneindex}}_{t}=1\;\&\\ -\frac{x_{j}}{L_{2}}, & j\_{\mathrm{laneindex}}_{t-1}=2\;or\;3\\ & j\_{\mathrm{laneindex}}_{t-1}=2\;or\;3\;\&\\ \frac{L_{2}-x_{j}}{L_{2}}, & j\_{\mathrm{laneindex}}_{t}=2\;or\;3 \end{array}\right. \end{array}$$
  where $D_{rh0\_0}$ denotes the sum of traffic densities on the ramp lane and the mainline lane 0; $D_{\mathrm{average}0}$ denotes the average traffic density estimated by summing the densities of all lanes and dividing by three, serving as the equivalent average density after merging four lanes into three; $L_{1}$ and $L_{2}$ represent the lengths of the Pre-Merging Zone and the Merging Zone, respectively; $x_{i}$ and $x_{j}$ denote the longitudinal positions of vehicles i and j, respectively; $i\_{\mathrm{laneindex}}_{t}$ and $j\_{\mathrm{laneindex}}_{t}$ indicate the lane indices of vehicles *i* and *j* at time step *t*; $j\_{\mathrm{laneindex}}_{t}$ denotes the vehicle type of the *j*-th vehicle; and cav_mg indicates a CAV on the ramp.
  To better illustrate the above reward design, Figure 2 provides a visual explanation.

- $R_{v}$ denotes the speed reward, defined as the normalized average speed of all vehicles. It is used to measure the overall traffic flow efficiency, aiming to improve the system’s average travel speed and throughput. The specific formulation is given by
  (9) $$R_{v}=\frac{1}{n}\sum_{i=1}^{n}\frac{v_{i}}{v_{\max}}$$
  where $v_{i}$ denotes the speed of the *j*-th vehicle, and $v_{max}$ represents the maximum speed limit for all vehicles, which is set to 60 km/h in this study.
- $P_{c}$, which denotes the collision penalty, defined as the total number of vehicle collision events occurring in the system at time *t*, is a penalty term that aims to suppress collision behavior. By imposing negative incentives on unsafe behavior, it guides intelligent agents to learn more stable and safer driving strategies, thereby improving the operational safety of the entire system during the ramp merging process.
- $P_{s}$ denotes the time-step penalty, which is a constant value of 1. This penalty item is intended to penalize inefficient behavior of intelligent agents during task execution. By imposing a fixed penalty on each uncompleted task time step, it guides intelligent agents to learn to complete merging tasks with higher timeliness during training, thereby improving overall decision-making efficiency.

#### 3.2.3. Action Space

At each decision-making time step, the agent computes the action to be executed by a CAV based on the currently observed environmental state. To accomplish the ramp merging task, the available actions for a CAV are defined as a discrete action set: $a_{i}=\left\{left,keep,right\right\}$, left denotes a lane change to the left, keep denotes keeping the current lane, and right denotes a lane change to the right. The action space of the agent is defined as $A={\left\{a_{i}\right\}}_{i=1}^{n}$, where *N* is the number of CAVs in the current scenario.

#### 3.2.4. KAN and Mutual Information

(1). Kolmogorov–Arnold Networks

As an alternative to the Multilayer Perceptron (MLP), the KAN [37] model is based on the Kolmogorov–Arnold representation theorem, which states that any multivariate function can be represented in the following form:

(10) $$f\left(x\right)=\sum_{i=1}^{2d+1}\Phi_{i}\left(\sum_{j=1}^{d}\phi_{ij}\left(x_{j}\right)\right)$$

where Φ and ϕ are both univariate functions.

**Figure 3:**
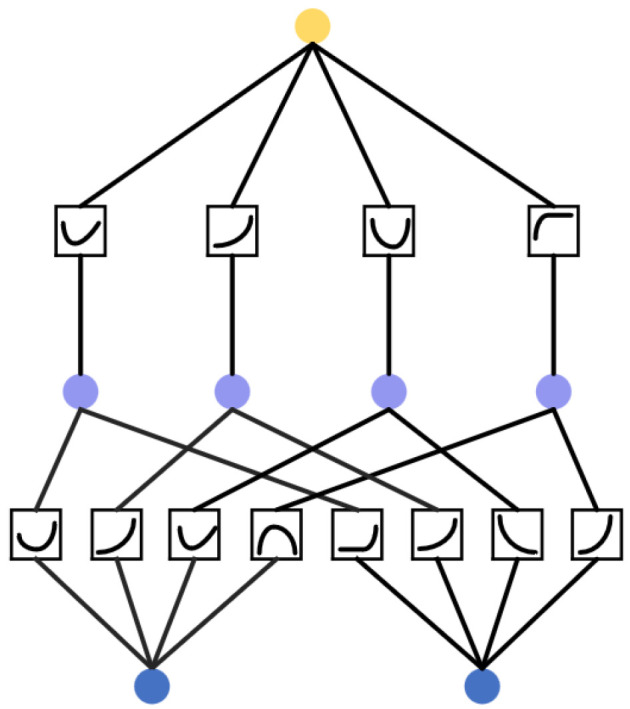
A two-layer KAN architecture.

In KAN, the activation functions are not located at the nodes but are instead distributed along the edges. These edge-based functions enable efficient function approximation and aggregation. This architecture explicitly incorporates the formulation given in Equation (10) into the network design. The construction ensures that all functions learnable by the network are compositions of univariate functions, which are further parameterized using B-spline functions. This significantly enhances the model’s capacity and flexibility in capturing nonlinear structures, thereby improving its overall feature learning ability. A two-layer KAN architecture is illustrated in Figure 3.

(2). Mutual Information

Mutual information (MI) [38] is a fundamental information-theoretic metric used to quantify the amount of information one random variable contains about another. It can also be interpreted as the reduction in uncertainty of one variable due to the knowledge of another. MI has been widely applied in various fields such as feature selection, neural network optimization, and GNN. Inspired by [39,40], a discriminator *D* is trained to maximize the mutual information between the embedded graph information *G* and the agent’s Q-value *Q*, thereby distinguishing between the positive sample pair (G,Q) and the negative sample pair $\left(\tilde{G},Q\right)$. Accordingly, the corresponding loss function is defined by

(11) $$L_{MI}=\log D\left(G,Q\right)+\log\left[1-D(\tilde{G},Q)\right]$$

## 4. Methodology

### 4.1. Method Overview

**Figure 4:**
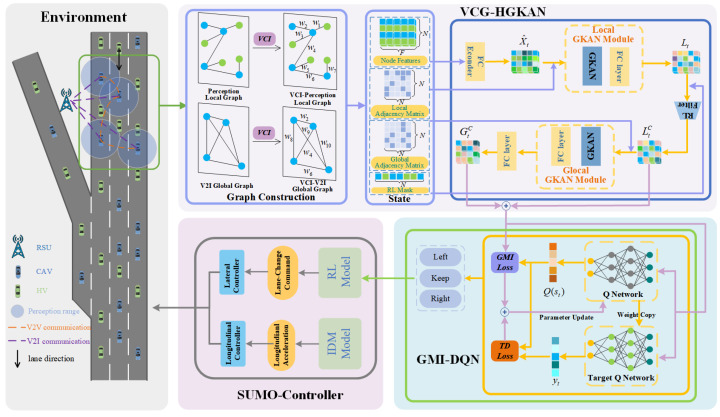
VCG-EGRL framework for ramp merging in mixed traffic.

To effectively capture the complex traffic states in mixed-traffic ramp merging areas and improve merging efficiency, traffic flow, and overall safety, this paper proposes a novel enhanced local–global GRL framework, as illustrated in Figure 4. The proposed framework consists of two core modules: a hierarchical graph network module (VCG-HGKAN) and an improved reinforcement learning-based action decision module. First, a local cooperative interaction graph is constructed based on the VCI model and vehicle perception–communication characteristics, and local features are extracted and aggregated using a local GKAN. Then, a global V2I communication graph is constructed based on V2I communication between CAVs and RSUs, and a global GKAN is applied to CAV nodes enriched with local information for deep feature extraction and integration. The resulting local and global features are concatenated to form comprehensive vehicle network features. Finally, the reinforcement learning decision module, trained under a graph mutual information maximization framework, learns the fused vehicle network features and generates joint actions for efficient multi-vehicle cooperation. This section will elaborate on the specific components of the proposed framework.

### 4.2. VCG-HGKAN Module

The VCG-HGKAN model mainly consists of two components: (1) a local–global graph structure constructed based on the VCI model and the V2V and V2I communication mechanisms, and (2) a feature extraction module based on the KAN-enhanced GCN (GKAN). The former is used to precisely model the complex local and global cooperative interactions among vehicles, while the latter enables effective representation and deep extraction of traffic environment features.

#### 4.2.1. Local–Global Graph Based on the VCI Model

This paper first proposes the VCI model to construct a cooperation intensity matrix among vehicles. This matrix is then integrated with vehicle perception capabilities and the V2V communication matrix to form the adjacency matrix of the local graph. The local graph is used to finely model the interaction relationships and state information between CAVs and their surrounding vehicles. The resulting local traffic environment features play a crucial role in supporting real-time decision-making of the model. Specifically, the local graph is defined as $G_{t}^{\mathrm{local}}=\left\{V_{t},E_{t}^{\mathrm{local}}\right\}$, where the node set $V_{t}=\left\{v_{t}^{i},\;i\in \left\{1,2,\ldots ,n\right\}\right\}$ represents the features of all vehicles in the ramp merging area at time *t*, and the edge set $E_{t}^{\mathrm{local}}=\left\{e_{t}^{ij},\;ij\in \left\{1,2,\ldots ,n\right\}\right\}$ encodes the cooperative intensity and perception–communication relationships among vehicles. Here, *n* denotes the number of CAVs within the ramp merging area.

The VCI model proposed in this paper is based on the Driving Safety Field (DSF) [41] to characterize the complex dynamic interactions between vehicles. The DSF model draws inspiration from the potential field theory in physics, using the concept of fields to quantify potential risks in the driving environment, while effectively representing the importance of surrounding vehicles to the self-vehicle and the strength of collaboration [42]. The model calculates the cooperation intensity between vehicles by integrating various dynamic factors during the driving process. In this study, the kinetic energy field and behavioral field are specifically used to determine the cooperation intensity between vehicles. The formulas for calculating the kinetic energy field and behavioral field between any two vehicles are given by

(12) $$E_{K}^{ji}=\frac{GR_{j}M_{j}}{d_{ji}}\exp\left(kv_{j}\right)$$

(13) $$E_{B}^{ji}=r_{j}E_{K}^{ji}$$

where $E_{K}^{ji}$ and $E_{B}^{ji}$ denote the kinetic field intensity and behavioral field intensity exerted by vehicle *j* on vehicle *i*, respectively. Based on the above field intensities, the VCI is calculated as

(14) $$\begin{array}{cc} F_{ji} & =\left(E_{K}^{ji}+E_{B}^{ji}\right)R_{j}M_{i}\left(1+r_{j}\right)\exp\left(-kv_{i}\right)\\ \; & =\frac{GR_{j}R_{j}M_{j}M_{i}}{d_{ji}}\left(1+r_{j}\right)\left(1+r_{j}\right)\exp\left(kv_{j}-kv_{i}\right) \end{array}$$

where *G* and *k* are predefined constants; $R_{i}$ and $R_{j}$ represent the road condition factors for vehicles *i* and *j*, respectively; $M_{i}$ and $M_{j}$ denote the masses of vehicles *i* and *j*; $r_{i}$ and $r_{j}$ are the risk factors associated with the driving styles of vehicles *i* and *j*; $v_{i}$ and $v_{j}$ are the velocities of vehicles *i* and *j*; and $d_{ji}$ is the distance between vehicle *i* and vehicle *j*.

Based on the obtained vehicle states, the cooperative intensity between all vehicle pairs can be calculated using the above formulas, thereby constructing the vehicle cooperation intensity matrix. To ensure the comparability of cooperation intensities across different vehicle pairs and to facilitate the construction of the subsequent local adjacency matrix, the values of $F_{ji}$ are normalized using min-max normalization:

(15) $$F_{ji}^{\prime }=\frac{F_{ji}-\min_{ji}F_{ji}}{\max_{ji}F_{ji}-\min_{ji}F_{ji}}$$

During the normalization process, most constant terms are canceled out. Therefore, the above cooperation intensity formula can be further simplified, from which the VCI matrix can be derived:

(16) $$F_{ji}=\frac{\exp(kv_{j}-kv_{i})}{d_{ji}}$$

In this paper, the parameter *k* is set to 0.05. In addition, to eliminate self-interference, the cooperation intensity of a vehicle with itself is set to 0.

(17) $$F_{t}={\left\{F_{ji}^{\prime }\right\}}_{i,j=1}^{n}$$

Equations (12)–(17) collectively define the vehicle collaboration intensity, which serves as the quantitative foundation for constructing both local and global interaction graphs in the proposed method.

CAVs are capable of actively perceiving the surrounding traffic environment through onboard sensors, enabling decision-making based on local traffic conditions (i.e., perception features). Meanwhile, CAVs can also exchange information over a broader spatial range via V2V communication to obtain more extensive awareness of traffic conditions (i.e., communication features). As illustrated in Figure 4, the blue circles and orange dashed lines represent the integrated interaction pattern of CAVs at the perception and communication levels. To model this integrated pattern, a perception–V2V communication matrix is constructed in this paper, where the edge weights represent the perception and communication relationships between vehicles. The edge connection between vehicle *i* and vehicle *j* is defined based on the perception capability and communication range included in the input features:

(18) $$s_{t}^{ij}=\left\{\begin{array}{cc} 1, & |p_{t}^{i}-p_{t}^{j}|\leq \rho \\ 0, & |p_{t}^{i}-p_{t}^{j}|>\rho \end{array}\right.$$

where $p_{t}^{i}$ and $p_{t}^{j}$ denote the positions of vehicles *i* and *j* at time *t*, respectively. ρ denotes the predefined perception–communication distance threshold for CAVs. In this paper, the perception range is set to ρ=20 m, and the V2V communication range is set to ρ=100 m. Based on $s_{t}^{ij}$, the perception–communication matrix $D_{t}^{l}$ is constructed by

(19) $$D_{t}^{l}={\left\{s_{t}^{ij}\right\}}_{i,j=1}^{N}$$

On this basis, by combining $F_{t}$ and $D_{t}^{l+}$, the local graph adjacency matrix $A_{t}^{\mathrm{local}}$ can be obtained. Its construction method is defined by:

(20) $$A_{t}^{\mathrm{local}}=F_{t}⊗D_{t}^{l^{\prime }}$$

where ⊗ denotes the element-wise Hadamard product, which is used to combine the perception–communication relationships with the cooperation strength matrix, enabling accurate construction of effective connectivity between nodes in the local interaction graph.

CAVs can communicate with roadside infrastructure (e.g., RSUs) via V2I communication to obtain the status information of other CAVs within the region. This allows CAVs to make decisions based on a broader scope of global traffic information. Such perception–communication fusion is beneficial for achieving long-term cooperative strategies and enhancing decision-making participation. As shown in Figure 4, the environment includes a predefined set of infrastructure nodes that support V2I communication. Using this communication mechanism, a global graph based on V2I communication is constructed to represent the global cooperative relationships among vehicles. The global graph is defined as $G_{t}^{\mathrm{global}}=\left\{V_{t},E_{t}^{\mathrm{global}}\right\}$, where $V_{t}$ represents the set of vehicle nodes in the global scope at time *t*, and $E_{t}^{\mathrm{global}}$ denotes the set of global communication edges based on the V2I communication mechanism. The edge relationships between vehicles i and j can be defined according to the vehicle type as

(21) $$c_{t}^{ij}=\left\{\begin{array}{cc} 1, & i\_type=j\_type=\mathrm{CAV}\\ 0, & \mathrm{else} \end{array}\right.$$

where i_type and j_type denote the vehicle types of vehicles *i* and *j*, respectively. Based on the defined edge relationships, the perception–communication matrix $D_{t}^{s}$ is constructed by

(22) $$D_{t}^{s}={\left\{c_{t}^{ij}\right\}}_{i,j=1}^{N}$$

On this basis, by combining $F_{t}$ and $D_{t}^{s}$, the global graph adjacency matrix $A_{t}^{\mathrm{global}}$ can be obtained. Its construction method is defined by

(23) $$A_{t}^{\mathrm{global}}=F_{t}⊗D_{t}^{s}$$

Equations (18)–(23) jointly define the construction of the vehicle collaboration graphs at both local and global levels. Specifically, the vehicle collaboration intensity is used to determine the edge weights between vehicles, while the connectivity patterns are established by incorporating V2I and V2V communication mechanisms, resulting in the local and global adjacency matrices. These graph structures explicitly encode interaction-aware cooperation relationships and serve as the input graphs for the subsequent GKAN-based feature extraction module.

#### 4.2.2. KAN-Enhanced GCN

In mixed-traffic ramp merging scenarios, various types of traffic participants coexist, including ramp CAVs, ramp HDVs, mainline CAVs, and mainline HDVs. The coexistence of these heterogeneous agents leads to highly complex and diverse interaction behaviors among vehicles. Moreover, vehicle motion patterns exhibit significant randomness and nonlinearity. Traditional GCN models struggle to effectively capture and represent the nonlinear and high-dimensional interactions inherent in such multi-agent traffic environments. Therefore, there is an urgent need to develop an improved GCN with enhanced representational capacity to more effectively model and capture the complex and nonlinear interaction features in mixed-traffic scenarios.

**Figure 5:**
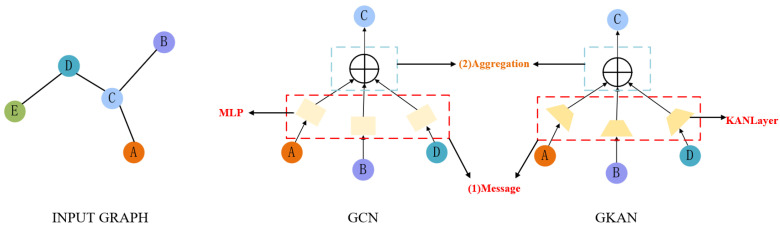
Message passing frameworks of GCN and GKAN.

GCN models are typically composed of multiple message-passing layers, each involving the update of node representations and the aggregation of information from neighboring nodes. In standard GCNs, the node representation update is usually achieved through a linear transformation followed by a non-linear activation function. Some variants employ MLP to enable more complex non-linear transformations. However, due to structural limitations, even with MLP, GCNs still face challenges in capturing highly complex nonlinear relationships between nodes. To address this issue, this paper proposes replacing the traditional GCN with a GKAN, where the core idea is to substitute the MLP components with KAN layers to enhance the nonlinear feature extraction capability of node representations. A comparison between the GKAN architecture and conventional GCN is illustrated in Figure 5. Specifically, the node update rule of GKAN can be expressed as

(24) $$Z_{t}=f_{\mathrm{KAN}}\left(A_{t},H_{t}\right)={\tilde{D}}_{t}^{-\frac{1}{2}}{\tilde{A}}_{t}{\tilde{D}}_{t}^{-\frac{1}{2}}\mathrm{KAN}\left(H_{t}\right)+b$$

where KAN() denotes the KAN layer.

#### 4.2.3. Theoretical Analysis of the VCG-HGKAN Module

First, the initial vehicle feature matrix $X_{t}$, as defined in Section 3.2.1, is high-dimensional information and needs to be processed through a fully connected (FC) layer. This dimensionality reduction yields a more representative low-dimensional vehicle feature matrix ${\hat{X}}_{t}$. The resulting low-dimensional vehicle features and the local graph adjacency matrix $A_{t}^{\mathrm{local}}$ are fed into the local GKAN module, enabling each CAV to perceive nearby cooperative interactions and obtain an accurate local traffic representation. This results in node embeddings $L_{t}^{i}$ (including both CAVs and HDVs). The formulation is given by

(25) $$L_{t}^{i}=f_{\mathrm{KAN}}\left(A_{t}^{\mathrm{local}},{\hat{X}}_{t}\right)={\tilde{D}}_{t}^{-\frac{1}{2}}{\tilde{A}}_{t}^{\mathrm{local}}{\tilde{D}}_{t}^{-\frac{1}{2}}\mathrm{KAN}\left({\hat{X}}_{t}\right)+b$$

Since this study focuses solely on controlling the behavior of CAVs, it is necessary to further filter the node embedding features output by the local GKAN to retain only the information related to CAVs. Specifically, this filtering is achieved by performing a Hadamard product between a mask matrix $M_{t}$ and the node embedding matrix $L_{t}$:

(26) $$L_{t}^{C}=M_{t}⊗L_{t}$$

Then, the CAV-only node embeddings $L_{t}^{C}$, which have aggregated information from surrounding vehicles, together with the global graph adjacency matrix $A_{t}^{\mathrm{local}}$, are fed into the global GKAN module. This allows each CAV to obtain global traffic state information within the ramp merging area. The resulting node embeddings with global latent features are denoted as $G_{t}^{C}$. The formulation is given by

(27) $$\begin{array}{cc} G_{t}^{C} & =f_{KAN}\left(A_{t}^{global},L_{t}^{C}\right)\\ \; & ={\tilde{D}}_{t}^{g^{-\frac{1}{2}}}A_{t}^{global}{\tilde{D}}_{t}^{g^{-\frac{1}{2}}}\mathrm{KAN}\left(L_{t}^{C}\right)+b \end{array}$$

Then, the local node embeddings $L_{t}^{C}$ obtained from the VCG-HGKAN module and the global node embeddings $G_{t}^{C}$ are concatenated to fuse both local and global information. The resulting comprehensive node embeddings, which incorporate both local and global traffic information, are denoted as $LG_{t}^{C}$.

#### 4.2.4. Improved Reinforcement Learning Framework

Finally, the fused local and global information of the CAVs, represented by the node embeddings $L_{t}^{C}$, is fed into the action decision module $D_{a}$ to evaluate the value of each action under a specific environmental state. These values quantify the advantage of each action and serve as the basis for decision-making by the intelligent agent. All the neural network modules constructed in this study, including the fully connected layer, the local–global hierarchical graph neural network (HGKAN), and the Q-network, can be uniformly represented as a network ${\hat{Q}}_{\theta }$, where θ represents the set of all weights. The output of this network corresponds to the Q-values for all possible actions in the action set and is given by

(28) $${\hat{Q}}_{\theta }\left(s_{t},a_{t}\right)=D_{a}\left(LG_{t}^{C},a_{t}\right)$$

where $a_{t}$ denotes the set of all possible actions.

The model is trained based on Q-learning, where the optimization process is realized through experience replay and synchronization between the evaluation network and the target network. During the interaction between the agent and the environment, each step generates a transition tuple $\left(s_{t},a_{t},r_{t},s_{t+1}\right)$, where $s_{t}$ is the current state, $a_{t}$ is the executed action, $r_{t}$ is the received reward, and $s_{t+1}$ is the next state. These interaction samples are stored in an experience replay buffer. During training, the system periodically samples random mini-batches from this buffer to update the model parameters. The core objective of training is to minimize the loss function and thereby optimize the network parameters. The loss function is defined by

(29) $$L_{\theta }=\frac{1}{b}\sum_{t}y_{t}-{\hat{Q}}_{\theta }\left(s_{t},a_{t}\right)$$

where *b* denotes the batch size, and $y_{t}$ is the target Q-value, calculated using the Bellman equation. The formulation is given by

(30) $$y_{t}=r_{t}+\gamma \max_{a}{\hat{Q}}_{\theta }\left(s_{t+1},a\right)$$

To further enhance GKAN’s ability to learn from local–global graphs in GRL and ensure that the obtained graph embeddings contain more information relevant to Q-value estimation, a graph mutual information loss function is designed based on the mutual information between the graph embeddings $LG_{t}^{C}$ and the output Q-values, as defined in Equation (11). The formulation is given by

(31) $$L_{GMI}=\log D\left(LG_{t}^{C},Q\right)+\log\left[1-D({\tilde{LG}}_{t}^{C},Q)\right]$$

where ${\tilde{LG}}_{t}^{C}$ is a negative sample of $LG_{t}^{C}$, generated by randomly perturbing $LG_{t}^{C}$. *D* denotes the discriminator function. In this paper, a bilinear layer is used as the discriminator. The computation is given by

(32) $$D\left(LG_{t}^{C},Q\right)=\sigma \left({\left(LG_{t}^{C}\right)}^{⊤}WQ\right)$$

where *W* is a learnable weight matrix, and σ is the sigmoid activation function.

Finally, the above graph mutual information loss is combined with the original DQN loss to form the complete training loss function *L*:

(33) $$L=\alpha L_{Q}+\beta L_{GMI}+\mu {∥\omega ∥}^{2}$$

where α and β are weighting coefficients used to balance the relative importance of the two loss components. ω represents all trainable parameters in the model, and μ is the L2-regularization coefficient.

In summary, This section presents a complete methodological pipeline of the proposed VCG-EGRL framework. The introduced formulations jointly define the vehicle collaboration intensity, the construction of local and global collaboration graphs, and the GKAN-based feature extraction and reinforcement learning process. This section focuses on the algorithmic design and practical implementation of the proposed method, which is subsequently validated through extensive experimental evaluation.

## 5. Simulation Results

### 5.1. Experimental Setup

#### 5.1.1. Simulation Parameters

**Figure 6:**
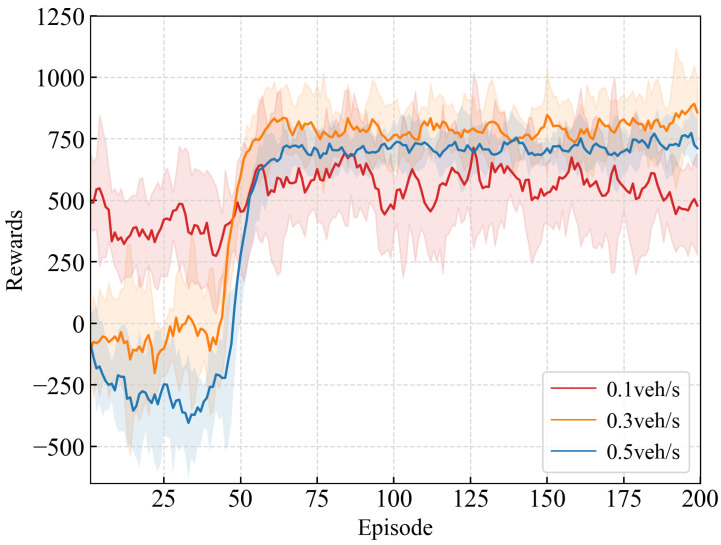
Training reward and step cost curves for each model. The solid line represents the mean value, and the shaded area indicates the corresponding standard deviation.

The ramp merging scenario adopted in this paper is shown in Figure 1. The maximum number of vehicles *N* is set to 56, with the maximum number of CAVs and HDVs both set to 28. The maximum speed of both ramp vehicles and main road vehicles is set to 60 km/h. At the start of the simulation, all vehicles enter the scene from the left side according to the pre-set traffic flow, and the positions and speeds of all vehicles are randomly generated. During the training process, the inflow rate of ramp vehicles is set to 0.3 veh/s, and the traffic flow rate of mainline vehicles is also set to 0.3 veh/s. To justify the parameter selection and analyze its sensitivity, comparative training experiments were conducted under different inflow rates (0.1/0.3/0.5 veh/s), and the corresponding learning curves are shown in Figure 6. When the inflow rate is 0.1 veh/s, sufficient available gaps exist in the merging area, resulting in limited inter-vehicle interactions and a low proportion of conflict samples. Consequently, the training signals are relatively sparse, the achieved rewards remain low, and the policy struggles to adequately learn representative merging game behaviors. In contrast, at inflow rates of 0.3 veh/s and 0.5 veh/s, merging interactions occur more frequently, enabling the agent to learn effective decision-making strategies under conflict scenarios. Among these settings, the inflow rate of 0.3 veh/s yields more stable convergence and higher reward values. Considering both training stability and subsequent cross-inflow validation results, an inflow rate of 0.3 veh/s is selected as the default parameter during training to characterize typical on-ramp merging conditions under moderate traffic demand.

#### 5.1.2. Training Parameters

The training parameters for the DRL model are set as follows: the total number of training episodes is 200, and the maximum number of steps per episode is 2500. If the step limit is reached, the episode terminates and the next one begins. The warm-up phase consists of $6\times 10^{4}$ steps, during which actions are selected entirely through ε-greedy random exploration. The exploration rate is set to 0.5. The size of the experience replay buffer is set to $10^{6}$. During the formal training phase, mini-batches of 32 transitions are randomly sampled from the buffer and used to update the network. The Adam optimizer is used with an initial learning rate of $\eta =1\times 10^{-4}$, and the discount factor is set to γ=0.9. The target Q-network is updated using a soft update rate of τ=0.1. The total loss function combines the DQN loss and the mutual information loss, with their respective weights set to α=1, β=0.3, and an L2 regularization coefficient of μ=0.01.

#### 5.1.3. Baseline Models

To verify the effectiveness of the proposed model, it is compared with three baseline models.

- Rule-Base: The built-in rule-based control algorithms in the SUMO simulator are adopted, including the IDM for longitudinal speed control and the LC2013 model for lateral lane-changing decisions.
- GCQ [29]: This model represents the traffic scenario as graph-structured data for the first time and adopts a GCN as the vehicle information fusion module. It is combined with a DQN equipped with an experience replay mechanism to perform reinforcement learning training for optimizing vehicle control strategies.
- GCAV-CPO [2]: This model is the first to integrate the vehicle graph structure with a multi-agent reinforcement learning framework, and proposes a distributed reinforcement learning approach for coordinating the cooperative merging decision-making and control of CAVs in multi-lane scenarios under mixed-traffic environments.

#### 5.1.4. Evaluation Metrics

In this paper, six metrics are used to evaluate the performance of the model: (1) Average reward: the reward averaged over all tested episodes. (2) Success rate: a merge is considered successful if the ramp vehicle completes the merging task without any collision. (3) Completion time: the average time taken by the CAVs to complete the on-ramp merging task. (4) Merge position: the average longitudinal position of the ramp vehicles when they successfully merge into the main roadway. (5) Average Potential Conflict Merging Rate (Average CPMR): the proportion of ramp vehicles in the merging area that experience potential conflict merging events. A potential conflict merging event is defined as a situation in which, at the decision-making instant of the ramp vehicle during merging, there exists a cross-lane interaction with the nearest mainline vehicle in the longitudinal direction, and the longitudinal distance between the two vehicles is smaller than a predefined threshold $s_{\min}$, which is given by $s_{\min}=s_{0}+v\left(t\right)\tau_{\min}$, where $s_{0}$ denotes the static minimum safe distance (set to 2m), and $\tau_{\min}$ represents the minimum safe time gap (set to 1s). (6) Unsafe TTC Percentage (TTC < 2 s): the proportion of simulation time steps during which the system is in a hazardous TTC state, defined as time-to-collision values smaller than 2 s.

**Figure 7:**
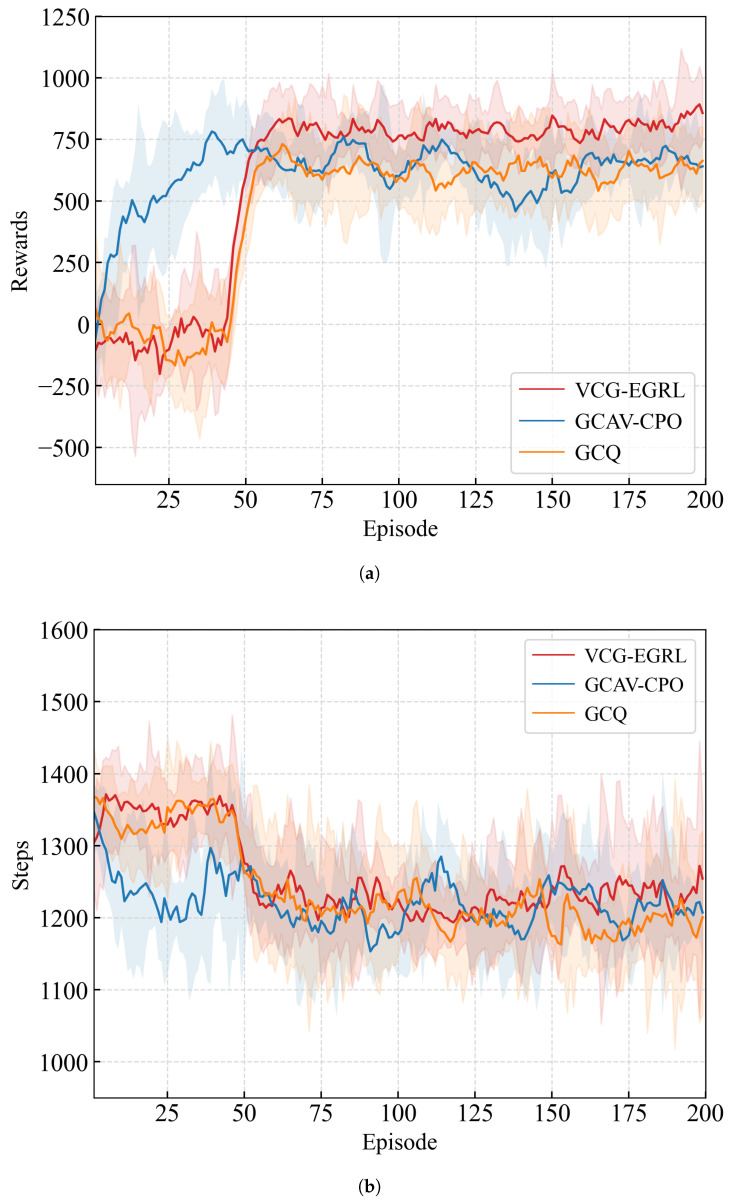
Training reward and step cost curves for each model. (**a**) Training reward curve. (**b**) Step cost curve.

Figure 7a illustrates the reward curve during model training, where the solid line represents the average value and the shaded area indicates the corresponding standard deviation. During the training process, the first $6\times 10^{4}$ steps (approximately 40 episodes) constitute the random exploration phase, in which CAVs execute actions randomly to explore the environment. As a result, the reward curve exhibits low-level fluctuations during this phase. From step $6\times 10^{4}$ to approximately $1.5\times 10^{5}$, the model gradually converges. During this period, the parameters of the GKAN module in VCG-EGRL are optimized, enabling the model to achieve a higher reward level. After the random exploration phase, the reward values obtained by the VCG-EGRL model are higher than those of the GCQ and GCAV-CPO models. As shown in the training step curve in Figure 7b, the time-step cost per episode gradually decreases as training progresses, demonstrating the effectiveness of the time-step penalty function during training.

### 5.2. Analysis of the Mode of Learned Policy

**Figure 8:**
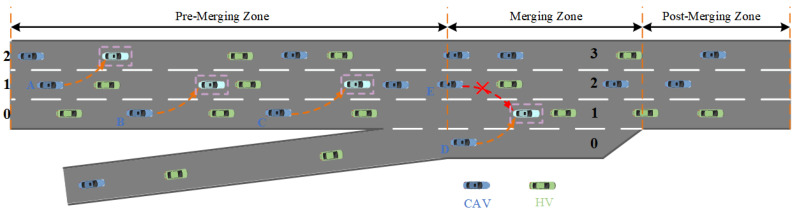
The illustration of learned policy.

The DRL models in this study are trained using the reward function defined in Section 3.2.2 to learn optimal policies. As shown in Figure 8, the learned strategies of the reinforcement learning models, including VCG-EGRL, are visualized. Lane indices 0, 1, 2, and 3 represent different lanes, with indexing differing between the Pre-Merging Zone and the Merging Zone. Vehicles within purple dashed boxes indicate the post-lane-change positions of CAVs. At the start of each simulation episode, CAVs and HDVs on both the ramp and mainline enter the network from the left with randomly assigned positions and speeds.

The control objective of the agent is as follows: when the combined vehicle density of the on-ramp and mainline lane 0 exceeds the overall average density, the CAVs on mainline lane 0 within the Pre-Merging Zone (e.g., vehicles B and C) should change to lane 1 as quickly and safely as possible before entering the merging area, in order to create more available merging space for the on-ramp vehicles. In the Pre-Merging Zone, the CAVs on mainline lanes 1 and 2 (e.g., vehicle A) will perform appropriate lane changes to either improve the overall traffic efficiency of the segment or provide lane-changing space for CAVs on lane 0, thereby assisting their lane-changing maneuvers. When the combined vehicle density of the on-ramp and mainline lane 0 is lower than the overall average density, CAVs on the mainline in the Pre-Merging Zone perform appropriate lane changes to improve the overall traffic efficiency of the segment. Then, in the Merging Zone, CAVs on lane 0 complete the on-ramp merging task by executing left lane changes as quickly as possible while ensuring safety (e.g., vehicle D). Throughout the entire process, CAVs on mainline lanes 1 and 2 in the Pre-Merging Zone are restricted from changing to lane 0, and CAVs on lanes 2 and 3 in the Merging Zone are restricted from changing to lane 1 (e.g., vehicle E). Simulation results demonstrate that the reinforcement learning agent prioritizes the safe and efficient completion of the on-ramp merging task by CAVs, with the learned policy aligning well with the designed reward function.

### 5.3. Analysis of Testing Results

After training is completed, the proposed method is comprehensively evaluated by testing both the proposed model and the baseline models under varying CAV penetration rates and traffic densities. To reduce the impact of randomness, all models are subjected to 50 independent test runs under identical experimental settings.

#### 5.3.1. Evaluation Under Different CAV Penetration Rates

**Figure 9:**
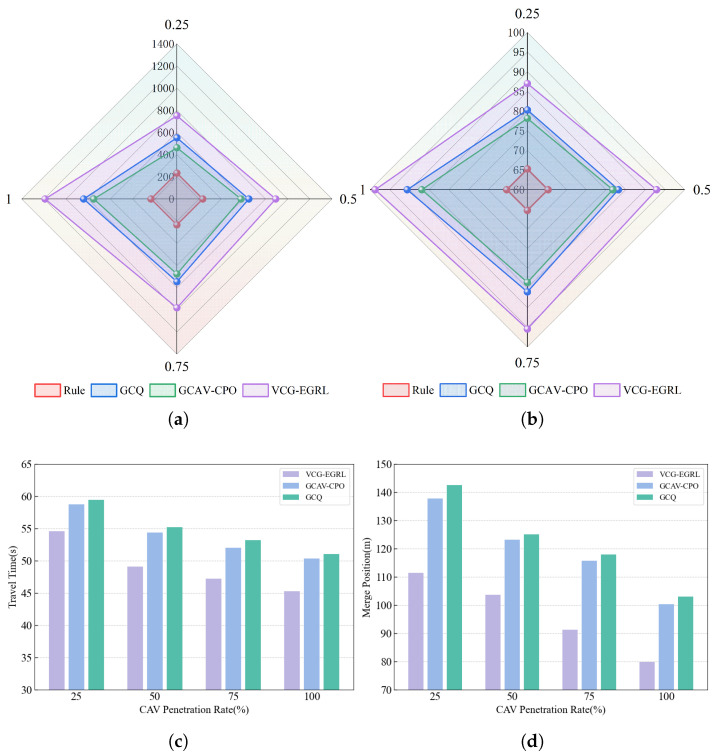

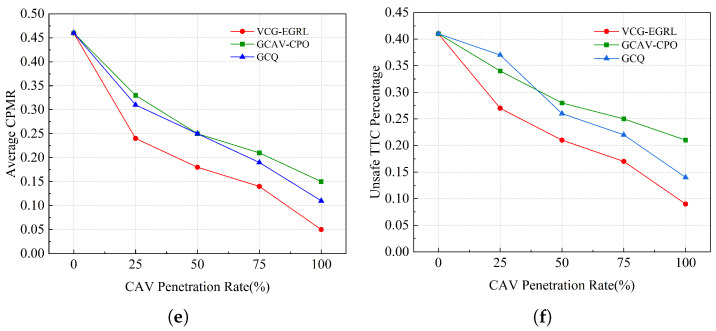
Sensitivity analysis of CAV penetration rates on test performance. (**a**) Mean reward. (**b**) Success rate. (**c**) Travel time. (**d**) Merge position. (**e**) Average CPMR. (**f**) Unsafe TTC Percentage.

To further evaluate the effectiveness of the proposed model, we tested the performance of VCG-EGRL, GCAV-CPO, and GCQ under different CAV penetration rate conditions. The test results are shown in Figure 9. As the CAV penetration rate increases, the number of CAVs on the main road also increases, thereby enhancing their active collaboration capabilities during the merging process. Main road CAVs tend to initiate lane changes earlier in the merge preparation zone, providing sufficient merging space for ramp vehicles. Therefore, as the penetration rate increases, the merging success rate of ramp vehicles increases, and the merging position becomes more forward (i.e., they can merge into the main road faster). This indicates that the introduction of CAVs facilitates the efficient completion of ramp merging tasks. Additionally, the merging success rate of ramp vehicles is positively correlated with the average reward, indicating that the reward function design is conducive to ramp merging. Compared with GCAV-CPO and GCQ, VCG-EGRL has higher rewards and success rates, while achieving lower merging positions and travel times. Therefore, the VCG-EGRL model can more efficiently complete ramp merging tasks. As illustrated in Figure 9e,f, the safety risks of the compared methods are evaluated under different CAV penetration rates from two perspectives: the Average CPMR and the Unsafe TTC Percentage. Overall, as the CAV penetration rate increases, both the Average CPMR and the Unsafe TTC Percentage of all three methods exhibit a monotonic decreasing trend. This indicates that higher CAV penetration effectively reduces close-range cross-lane interactions and near-miss risks during the merging process. Consequently, the need for forced braking by mainline vehicles is alleviated, which helps mitigate mainline traffic disturbances and lowers the potential risk of traffic accidents. VCG-EGRL consistently achieves lower Average CPMR and Unsafe TTC Percentage across all penetration levels, with more pronounced advantages in the medium-to-high penetration range. This suggests that VCG-EGRL is more inclined to select safer gaps and suppress high-risk merging behaviors at critical merging moments, thereby reducing the impact on mainline traffic while maintaining merging feasibility. In contrast, although the risks associated with GCAV-CPO and GCQ also decrease as the penetration rate increases, these methods still exhibit higher potential conflict and TTC risks at most penetration levels. This reflects their relatively conservative or less stable performance in gap utilization and risk control during the merging process.

#### 5.3.2. Robustness Evaluation Under Different Traffic Densities

**Figure 10:**
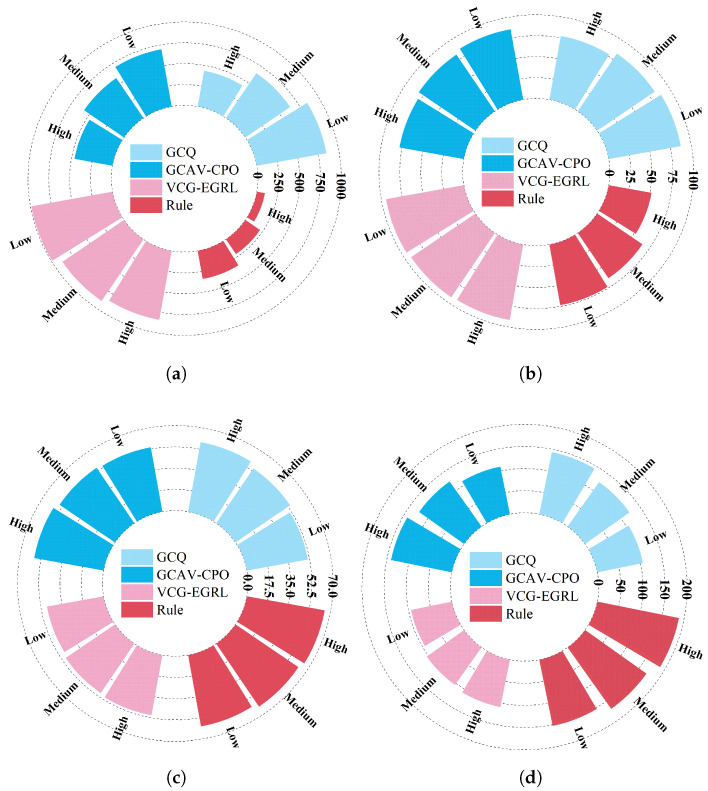
Performance of different models under different traffic densities. (**a**) Mean reward. (**b**) Success rate. (**c**) Travel time. (**d**) Merge position.

To assess the robustness of the proposed model under different traffic load conditions, this study conducted systematic tests on the VCG-EGRL model and three baseline models under three main road traffic density settings. During the testing process, the CAV penetration rate was fixed at 50%, the inflow rate of vehicles on the ramp was fixed at 0.3 veh/s, and the inflow rates of vehicles on the main road were set to 0.1 veh/s (Low), 0.3 veh/s (Medium), and 0.5 veh/s (High), corresponding to the construction of three simulation scenarios with different traffic densities. Under each traffic density setting, 50 simulation rounds were run and the average values of various evaluation indicators were calculated. The test results are shown in Figure 10. The three reinforcement learning models performed better in environments with low vehicle inflow rates on the main road. This may be attributed to the fact that in scenarios with high vehicle inflow rates on the main road, vehicle density increases and the available merging gaps for ramp vehicles are significantly reduced, leading to increased difficulty in completing merging tasks. Additionally, in scenarios with high main road vehicle inflow rates, the complexity of environmental information increases, forcing agents to process more complex dynamic information at each step, thereby increasing the difficulty of decision-making. The VCG-EGRL, GCAV-CPO, and GCQ models demonstrate higher robustness than rule-based models in various scenarios, indicating the vulnerability of rule-based models in responding to environmental changes and highlighting the ability of GNN-based DRL methods to generate efficient and robust decisions. Further comparison reveals that the VCG-EGRL model exhibits the least performance fluctuation across different scenarios compared to the GCAV-CPO and GCQ models, demonstrating stronger robustness. This indicates that the GKAN in the VCG-EGRL model is better able to process complex information in high-traffic density scenarios, enabling it to make robust and efficient decisions in complex environments.

Table 1 presents a comparison of different methods with respect to two safety risk metrics, namely the Average CPMR and the Unsafe TTC Percentage, under different traffic density conditions (Low/Medium/High). Overall, as traffic density increases from Low to High, both the Average CPMR and the Unsafe TTC Percentage exhibit an increasing trend across all methods. This indicates that higher traffic demand significantly compresses available merging gaps and intensifies the complexity of vehicle interactions, thereby increasing the frequency of close-range cross-lane interactions and near-miss risks. As a result, mainline vehicles are more prone to braking and speed adjustment maneuvers, leading to amplified mainline disturbances and elevated potential accident risks. VCG-EGRL consistently achieves lower Average CPMR and Unsafe TTC Percentage under Low, Medium, and High traffic densities, demonstrating a clear and consistent advantage over GCAV-CPO, GCQ, and the rule-based method. Although the safety risk metrics of all methods increase with rising traffic density, the growth rate of VCG-EGRL is noticeably smaller, indicating its stronger risk mitigation capability under high-demand conditions. These results demonstrate that the proposed method effectively reduces potential conflict events and TTC-related risks across varying traffic densities, avoiding scenarios in which successful merging is achieved at the expense of elevated safety costs.

#### 5.3.3. Ablation Study

To further evaluate the contribution of each key module within the VCG-EGRL model to overall performance, a series of ablation studies were designed and conducted. The differences among the ablated models are summarized in Table 2.

The results are shown in Table 3. Compared with the GCQ model, the MI-EGRL model shows improvements in key metrics such as average reward value and merger success rate. This indicates that the GRL trained using a framework with a graph mutual information loss function can enhance the GNN’s ability to extract spatial driving features of vehicles, thereby supporting the model in making more accurate decisions. Compared with the GCQ model, the VCG-GRL model shows significant improvements in all evaluation metrics. This indicates that the local–global graph based on the VCI model can more clearly determine the collaborative vehicles within the local scope and the collaborative strength, more effectively encode the collaborative interaction effects between vehicles, and simultaneously utilize the global graph constructed by V2I communication to achieve perception of the overall traffic state. Combined with the improved graph neural network GKAN, it can more effectively extract the complex spatial driving features of collaborative vehicles, thereby improving the merging efficiency of ramp vehicles. Overall, the performance advantages of the VCG-EGRL model are attributed to the synergistic effects of its various submodules. This comprehensive and integrated design enables the model to better adapt to complex traffic environments and efficiently complete ramp merging tasks under mixed-traffic conditions.

To evaluate the computational cost and real-time feasibility of the proposed method, we compare the model size and inference latency of the baseline GCN-based model (GCQ) and the proposed GKAN-based framework (VCG-EGRL). The number of trainable parameters and the average inference latency per decision step are reported under the same experimental setting. As shown in Table 4, VCG-EGRL introduces a modest increase in model size, with the number of parameters increasing from 39,651 to 41,827. Due to the incorporation of B-spline-based edge interaction modeling, the average inference latency increases from 5.485 ms/step to 7.698 ms/step. Although additional computational cost and inference time are introduced, the proposed method still satisfies the real-time requirements of autonomous driving decision-making. These results demonstrate that the proposed GKAN-based framework achieves enhanced interaction representation capability while maintaining practical real-time feasibility.

**Figure 11:**
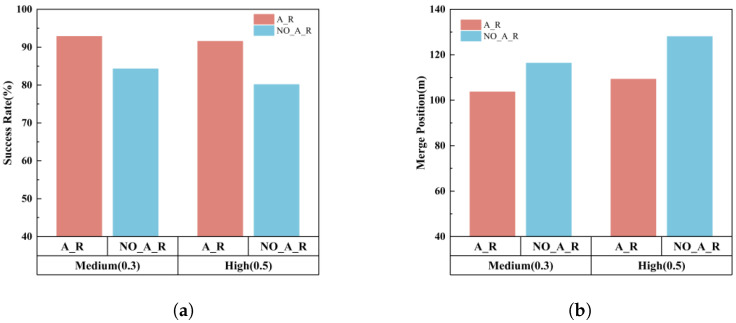
The impact of auxiliary merger rewards on merger tasks. (**a**) Success rate. (**b**) Merge position.

In this study, in addition to comparing the impact of different modules on the performance of the VCG-EGRL model, we further analyze the specific effect of the proposed merging reward design on the ramp merging task, as illustrated in Figure 11. The experimental setup includes two comparative models: NO_A_R, which refers to the model trained without the auxiliary merging reward for vehicles in the Pre-Merging Zone, and A_R, which represents the model trained under the complete reward framework.

By comparing the test results of the two models under the same mainline traffic density, it is observed that the model without the auxiliary merging reward exhibits a decline in both merging success rate and merging efficiency. This indicates that the auxiliary merging reward plays a critical role in enhancing the performance of the ramp merging task. Specifically, this reward component guides mainline vehicles to proactively change lanes in advance, thereby creating more available merging space. As a result, ramp vehicles are able to complete the merging task earlier, which significantly improves merging efficiency.

Further comparative analysis of the two models under different mainline traffic densities reveals that, under high mainline density conditions, the performance degradation in terms of merging success rate and merging efficiency is more pronounced when the auxiliary merging reward is removed. This result indicates that the auxiliary merging reward plays a more significant role in improving the overall system efficiency in complex traffic environments where congestion is likely to occur in the ramp merging area. By introducing this reward mechanism, mainline vehicles in lanes adjacent to the ramp are effectively guided to adjust their distribution in advance, thereby reducing local traffic density. This creates more favorable merging conditions for ramp vehicles, enabling them to complete merging more quickly and alleviating potential congestion risks in the Merging Zone.

## 6. Conclusions

To effectively model vehicle cooperative interaction relationships and comprehensively extract complex driving behavior features, this paper proposes a novel VCG-EGRL algorithm to address the multi-vehicle cooperative merging control problem in on-ramp merging areas under mixed-traffic conditions. The proposed algorithm embeds a hierarchical graph convolutional network into a deep reinforcement learning (DRL) framework, constructing a centralized decision-making model capable of handling a dynamic number of agents. Specifically, a local–global graph is constructed based on the VCI model, incorporating both vehicle perception–communication relationships and V2I communication links. GKAN is then utilized to process the local and global graphs separately, capturing complex driving features and cooperative interactions of vehicle nodes from both macro and micro perspectives. Finally, a DQN algorithm trained within a graph mutual information maximization framework is used to generate cooperative merging strategies for CAVs in the merging area. The proposed model is evaluated under various vehicle compositions and mainline traffic densities. Experimental results demonstrate that the proposed method significantly improves merging success rate and efficiency, while exhibiting strong robustness. Moreover, ablation studies confirm the effectiveness of the proposed auxiliary merging reward function.

## Figures and Tables

**Table 1:** Comparison of safety risk metrics under different traffic density conditions.

Traffic Density	Metric	VCG-EGRL	GCAV-CPO	GCQ	Rule-Base
Low	Average CPMR	0.08	0.13	0.10	0.25
	Unsafe TTC Percentage	0.11	0.17	0.14	0.22
Medium	Average CPMR	0.18	0.25	0.25	0.46
	Unsafe TTC Percentage	0.21	0.28	0.28	0.41
High	Average CPMR	0.29	0.37	0.35	0.59
	Unsafe TTC Percentage	0.34	0.40	0.39	0.51

**Table 2:** Configuration of different model variants for the ablation study. "—" indicates that the corresponding module is not included, while "*√*" indicates that the module is included.

Model	VCG-HGKAN	GMI Training Framework
GCQ	—	—
MI-EGRL	—	*√*
VCG-GRL	*√*	—
VCG-EGRL	*√*	*√*

**Table 3:** Comparison of different methods under multiple performance metrics.

Metric	GCQ	MI-EGRL	VCG-GRL	VCG-EGRL
Reward	648.87	722.37	780.42	892.06
Success Rate (%)	83.14	86.43	88.71	92.86
Travel Time (s)	54.37	53.30	52.02	49.12
Merge Position (m)	123.22	115.27	110.76	103.68
Average CPMR	0.25	0.22	0.21	0.18
Unsafe TTC Percentage	0.26	0.23	0.23	0.21

**Table 4:** Computational cost comparison.

Model	Parameters	Average Inference Latency (ms)
GCQ	39,651	5.49
VCG-EGRL	41,827	7.71

## Data Availability

The raw data supporting the conclusions of this article will be made available by the authors on request.
